# Tubular dysfunction in stable adult sickle cell patients in Kinshasa: prevalence and associated factors - a cross-sectional study

**DOI:** 10.1080/0886022X.2026.2702771

**Published:** 2026-08-03

**Authors:** Yannick Mompango Engole, Jean-Robert Rissassy Makulo, Yannick Mayamba Nlandu, Aliocha Natuhoyila Nkodila, Justine Busanga Bukabau, Vieux Momeme Mokoli, Marie-France Ingole Mboliasa, Augustin Luzayadio Longo, Blondy Mansoni, Rague Mvibudulu, Ernest Kiswaya Sumaili

**Affiliations:** a Nephrology Unit, Kinshasa University Hospital, University of Kinshasa, Kinshasa XI, Democratic Republic of the Congo; b Specialized Clinics of the Kinshasa, Kinshasa, Democratic Republic of the Congo; c SANRU, Kinshasa, Democratic Republic of the Congo

**Keywords:** Sickle cell disease, tubular dysfunction, α1-microglobulin, albuminuria, hydroxyurea

## Abstract

Renal complications of sickle cell disease (SCD) often begin with tubular dysfunction (TD), yet this entity remains insufficiently recognized in African populations. Data from adults in Kinshasa are particularly scarce. We conducted a cross-sectional study between March 2023 and April 2024, enrolling 279 clinically stable adult SCD patients from 14 centers in Kinshasa. Tubulopathy was defined by the concomitant presence of three abnormalities: urinary α1-microglobulin-to-creatinine ratio (A1M/Cr) ≥20 mg/g, dipstick glucosuria ≥1+ with plasma glucose <126 mg/dL, and urine pH ≥5.5. Glomerular involvement was assessed by urinary albumin to creatinine ratio (UACR ≥30 mg/g). Measured GFR (mGFR) was obtained by iohexol clearance. Multivariable logistic regression was used to identify independent factors associated with tubulopathy. Tubulopathy was identified in 58 patients (20.8%). Compared with those without tubulopathy, affected patients had significantly higher median A1M/Cr (44.7 vs. 14.2 mg/g; *p* < 0.001) and UACR (22.1 vs. 10.7 mg/g; *p* = 0.007). They also displayed lower mean eGFR by CKD-EPI_crea+cys2021_ (107.4 vs. 115.9 mL/min/1.73 m²; *p* = 0.031) and higher LDH levels (217.0 vs. 209.1 IU/L; *p* = 0.016). In multivariable analysis, independent predictors of tubulopathy included higher LDH (per 100 IU/L increase) (aOR 2.53, 95% CI 1.14–5.61), UACR ≥30 mg/g (aOR 1.94, 95% CI 1.18–5.18), while hydroxyurea use was independently associated with lower odds of tubulopathy (aOR 0.37, 95% CI 0.16–0.87). One in five stable adult SCD patients in Kinshasa presented with tubulopathy. This phenotype was associated with hemolysis, albuminuria, and lower eGFR, whereas hydroxyurea use was inversely associated with tubulopathy.

## PLAIN LANGUAGE SUMMARY

People living with sickle cell disease can develop kidney damage long before standard kidney tests become abnormal. One of the earliest changes affects the kidney tubules, which help the body conserve water and maintain the balance of salts and acids. Because these abnormalities are often overlooked, especially in African countries, we studied adults with sickle cell disease in Kinshasa, Democratic Republic of the Congo. We found that about one in five patients had evidence of tubular dysfunction despite preserved kidney filtration. Tubular dysfunction was more common in patients with higher levels of hemolysis and albuminuria, whereas patients receiving hydroxyurea were less likely to present with this phenotype. These findings support the importance of incorporating tubular biomarkers into the routine evaluation of sickle cell kidney disease and highlight the need for longitudinal studies to determine whether earlier detection can improve kidney outcomes.

## KEY MESSAGES

What is already known?

Tubular dysfunction is an early feature of sickle cell nephropathy but remains poorly characterized in African adults.

What does this study add?

One in five clinically stable adults with sickle cell disease had tubular dysfunction, independently associated with hemolysis, albuminuria, and lower eGFR.

What is the potential impact?

Tubular biomarkers may improve early detection of sickle cell nephropathy and guide future longitudinal studies.

Social Media “Hook”: One in five adults with sickle cell disease in Kinshasa had early tubular dysfunction despite preserved kidney function.

## Introduction

Sickle cell disease (SCD) is one of the most common haemoglobinopathies worldwide, with nearly 300,000 newborns affected each year, of which about 80% are born in sub-Saharan Africa [1,2]. In the Democratic Republic of Congo (DRC), the prevalence of the homozygous form (HbSS) is estimated at 2% of births, representing 30,000–40,000 new cases annually [3,4], underscoring the substantial burden of this disease.

Sickle cell nephropathy often begins early in life and spans a wide spectrum, from glomerular hyperfiltration and albuminuria to tubular dysfunction and ultimately end-stage renal disease [5]. Proximal tubular dysfunction is particularly insidious and frequently overlooked. It is characterized by increased urinary excretion of biomarkers such as α1-microglobulin, β2-microglobulin, or retinol-binding protein (RBP), which reflect impaired tubular reabsorption [6,7].

Pathophysiologically, recurrent medullary ischemia caused by vaso-occlusive crises (VOCs), glomerular hyperfiltration, oxidative stress, and the nephrotoxic effects of intravascular hemolysis (free hemoglobin, iron) all contribute to proximal tubular damage [5]. Emerging biomarkers such as cystatin C and N-acetyl-β-D-glucosaminidase (NAG) have been recognized as more sensitive markers for early detection of these alterations [7]. Chronic hemolysis releases cell-free hemoglobin and heme, driving nitric oxide depletion, oxidative stress, and microvascular dysfunction that preferentially affect the renal medulla and promote tubular ischemia [8]. Hemolysis-derived microparticles and extracellular vesicles further enhance inflammatory and procoagulant signaling [9], and may act as early triggers of vaso-occlusive and vascular injury, linking chronic hemolysis to early sickle cell–related tubulopathy before overt GFR decline [10].

Several studies have documented the high prevalence of renal involvement in SCD. Ranque et al. in a multicenter study conducted in Senegal, Cameroon, and Ghana, reported that 30–40% of adult patients presented with significant hyperfiltration or proteinuria [6,11]. In Saudi Arabia, Badr et al. demonstrated proximal tubular dysfunction in children with SCD, characterized by elevated urinary excretion of RBP and β2-microglobulin [12].

In the African context, although renal complications of SCD have been described in countries such as Senegal and Nigeria [6], data remain scarce regarding proximal tubulopathy in adult patients in the DRC, particularly in Kinshasa. Moreover, limited access to reliable biomarkers (α1-microglobulin, glucosuria, albumin/protein ratio) restricts early screening in resource-constrained settings. Therefore, the objective of this study was to determine the prevalence of tubulopathy and its associated factors in adult SCD patients followed in Kinshasa.

## Methods

This cross-sectional study was conducted between March 2023 and April 2024 among adults with sickle cell disease (SCD) in Kinshasa, Democratic Republic of Congo (DRC). Patients were recruited consecutively (convenience sampling) in stable clinical condition through collaboration with their primary healthcare providers across 14 centers, including Mixed Medical Center for Anemia SS, Makala General Reference Hospital, Bokoko Medical Center, Akram Hospital, Messie Center, Elisabeth Clinic, Amza Foundation, Rezodrepa, Codek, Colombe, Kalaki Foundation, and Lisungi. We excluded individuals with a history of hypertension, diabetes, or obesity, with urinary tract infection, menstruation, pregnancy as well as those presenting with vaso-occlusive crises, fever, acute chest syndrome, or recent use (within one month) of non-steroidal anti-inflammatory drugs (NSAIDs) or medications known to interfere with creatinine secretion (e.g. cimetidine, trimethoprim). Ethical approval was obtained from the Ethics Committee of the Faculty of Public Health, University of Kinshasa (approval no. ESP/CE/143/2022), and all participants provided written informed consent. Study results were forwarded to patients’ usual healthcare providers.

Clinical history and anthropometric data (weight, height, body fat percentage) were collected. Laboratory analyses included cystatin C, C-reactive protein (CRP), and ferritin (immunonephelometry, I-Chroma 2, Seoul, South Korea); serum creatinine (enzymatic method, Cobas, Roche Diagnostics, Germany); and complete blood count (impedance method, Zybio, Seoul, South Korea). Lactate dehydrogenase (LDH), aspartate aminotransferase (AST), and total/direct bilirubin were determined by kinetic methods on a semi-automated system. Hemoglobin S quantification was performed by cellulose acetate electrophoresis using the Gazelle device (Hemex Health, Portland, USA).

Measured GFR (mGFR) was determined from plasma iohexol clearance (Omnipaque 350 mg/mL; GE Healthcare, Belgium). After a 3.45 mL injection, 5 mL blood samples were collected at 60, 120, 180, 240, and 300 min from the contralateral arm. Samples were centrifuged and stored at 80 °C before shipment to the University of Liège (Belgium) [13]. Plasma iohexol concentrations were measured by liquid chromatography tandem mass spectrometry (LC-MS/MS) in an ISO 15189-accredited laboratory participating in external quality assessment (Equalis AB, Sweden) [14]. eGFR was estimated using the CKD-EPI_crea+cys2021_ (mixed) equation.

Urinary analyses were performed on first-morning spot urine samples. The urinary albumin-to-creatinine ratio (UACR) was calculated by dividing albumin (mg/L) by creatinine (mg/L), both measured by immunoturbidimetry (Cobas, Roche Diagnostics, Germany). The α1-microglobulin-to-creatinine ratio (A1M/Cr) was determined using the same method. Urinary pH and glucose levels were assessed with automated dipstick testing (Cypress Diagnostics, Belgium).

## Operational definitions

**Figure 1.**
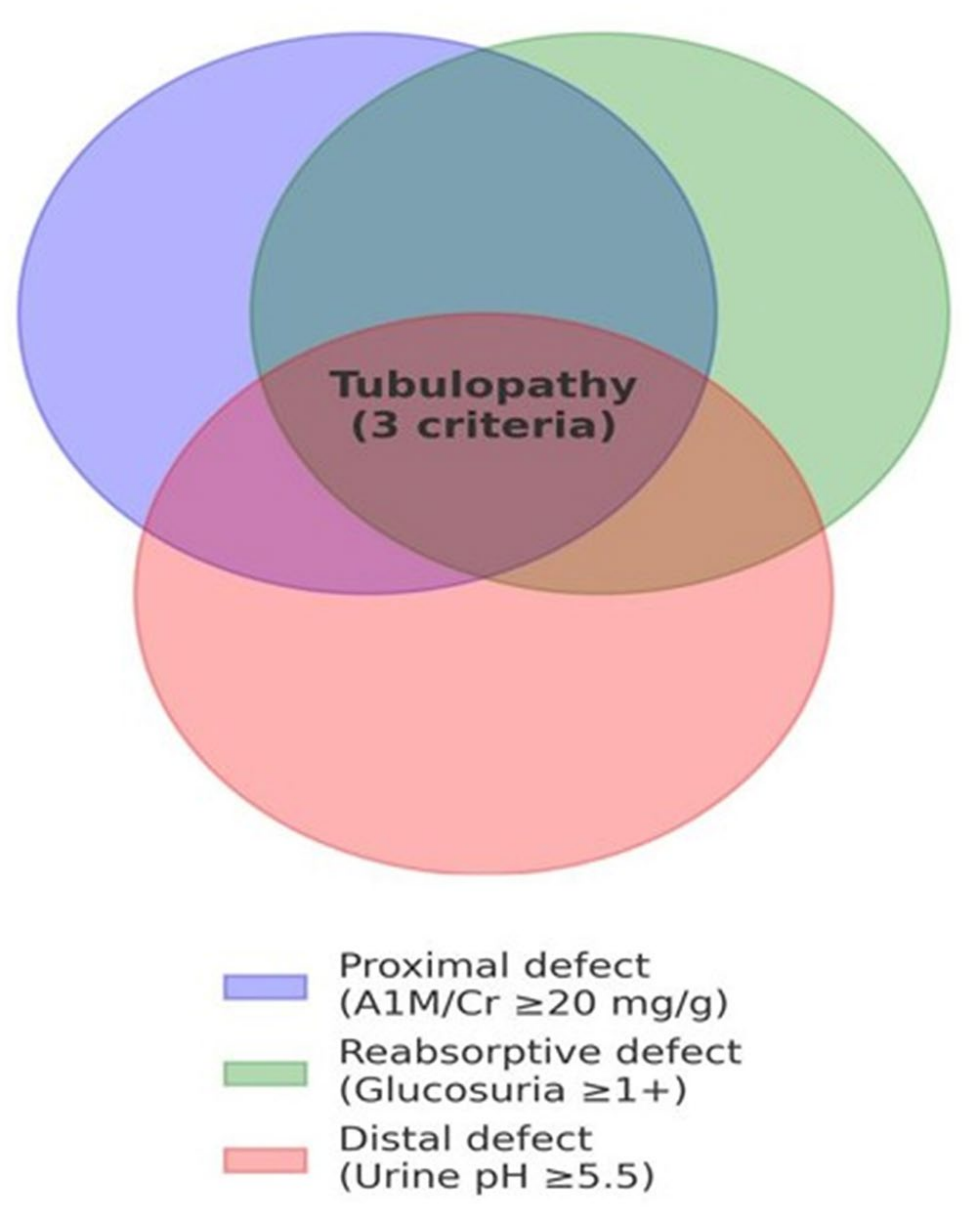
Tubulopathy composite definition.

- Tubulopathy was defined (Figure 1) by a stringent multi-marker approach, the concomitant presence of an A1M/Cr ≥20 mg/g, dipstick glucosuria ≥1+ with plasma glucose <126 mg/dL, and urinary pH ≥5.5 [15–17]. A first-morning urine pH ≥5.5 was included as an exploratory marker of urinary alkalinity that may reflect impaired distal urinary acidification. However, in the absence of metabolic acidosis or standardized acidification testing (e.g., furosemide–fludrocortisone or ammonium chloride loading), this parameter should not be interpreted as diagnostic of distal renal tubular acidosis
- Control group: Patients classified as having no tubulopathy were defined by the absence of all three tubular abnormalities used in the composite definition, namely: urinary α1-microglobulin-to-creatinine ratio <20 mg/g, absence of glucosuria on dipstick testing, and urinary pH <5.5.
- Glomerular involvement: UACR ≥30 mg/g [18]
- No glomerular involvement: UACR <30 mg/g.
- Mixed involvement: coexistence of tubulopathy and glomerular involvement.
- Stable SCD: absence of VOC, fever, or acute complications in the preceding month [19].

### Statistical analysis

Data were analyzed using SPSS v.25 (IBM Corp., Armonk, NY, USA). Continuous variables were expressed as mean ± standard deviation (SD) if normally distributed (Shapiro–Wilk test) or median [interquartile range, IQR] otherwise. Qualitative variables were summarized as counts (n) and percentages (%). Group comparisons were made using Student’s t-test or Mann–Whitney test for continuous variables, and chi-square or Fisher’s exact test for categorical variables as appropriate. Correlations between biomarkers urinary α1-microglobulin/creatinine, UARC and eGFR CKD-EPI creatinine-cystatine C 2021 were assessed with Spearman’s rho. Factors independently associated with tubulopathy were identified using multivariate logistic regression model (after adjustment of potential confounding factors), with results expressed as adjusted odds ratios (adjusted OR) and 95% confidence intervals (CI). LDH, AST, and eGFR (CKD-EPI creatinine–cystatin C 2021) were modeled as continuous variables. To improve the clinical interpretability of the regression coefficients, these variables were rescaled so that the odds ratios represented the change in the odds of tubulopathy associated with each 100 IU/L increase in serum LDH, each 10 IU/L increase in serum AST, and each 10 mL/min/1.73 m^2^ increase in eGFR. Statistical significance was set at *p* < 0.05.

## Results

**Figure 2.**
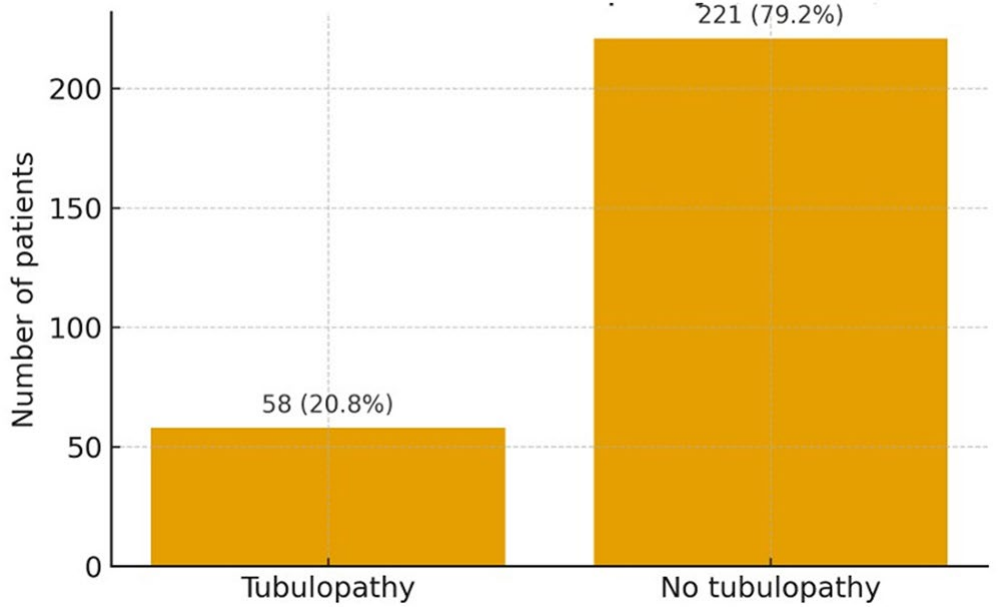
Prevalence of tubulopathy in the study population.

**Figure 3.**
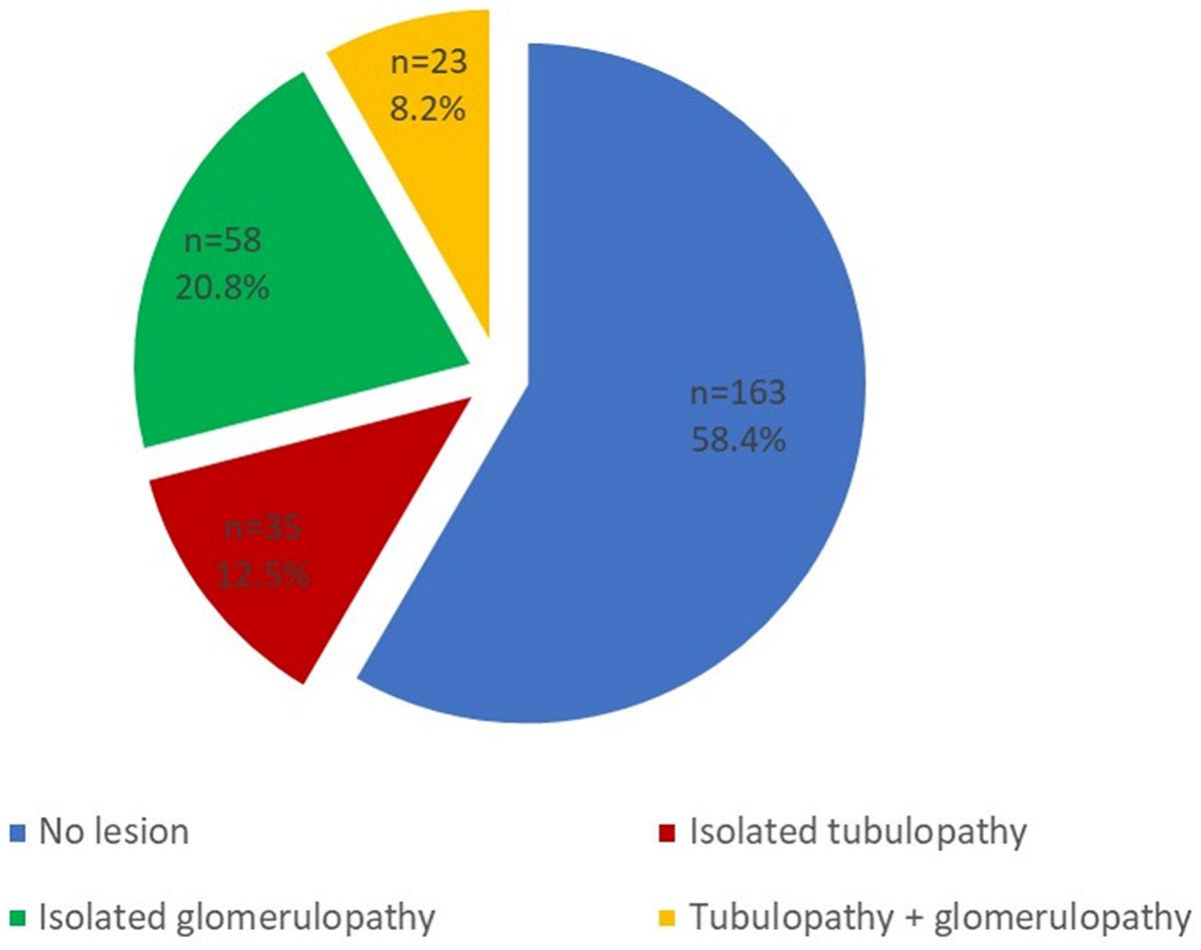
Prevalence of urinary abnormalities in the study population.

Among the 279 patients enrolled, tubulopathy was identified in 20.8% (*n* = 58) (Figure 2). Isolated tubulopathy was observed in 12.5% (*n* = 35), while isolated glomerular involvement occurred in 20.8% (*n* = 58) and combined tubulo-glomerular lesions in 8.2% (*n* = 23) (Figure 3). No significant differences were found between patients with and without tubulopathy regarding age, sex. However, tubulopathy was less frequently associated with retinopathy (28.1% vs. 5.2%; *p* = 0.008) and fetal hemoglobin (HbF) ≥ 15 (21.9 vs. 3.9%; *p* = 0.001), whereas the use of hydroxyurea was less common in this group (22.4 vs. 39.4%; *p* = 0.011) (Table 1).

**Table 1.** General characteristics of study population.

Variables	Entire population (*n* = 279)	No tubulopathy (*n* = 221)	Tubulopathy (*n* = 58)	*p*
Sex				0.355
Female	167 (59.9)	134 (60.6)	33 (56.9)	
Male	112 (40.1)	87 (39.4)	25 (43.1)	
Folic acid intake	254 (91.0)	203 (91.9)	51 (87.9)	0.243
Hydroxyurea intake	100 (35.8)	87 (39.4)	13 (22.4)	**0.011**
Surgery	66 (23.7)	50 (22.6)	16 (27.6)	0.265
FH-VOC	115 (41.2)	94 (42.5)	21 (36.2)	0.236
Leg ulcer	76 (27.2)	58 (26.2)	18 (31.0)	0.283
Priapism	17 (6.1)	13 (5.9)	4 (6.9)	0.486
Retinopathy	43 (15.4)	40 (18.1)	3 (5.2)	**0.008**
Aseptic necrosis of femoral head	51 (18.3)	41 (18.6)	10 (17.2)	0.494
Osteomyelisis	3 (1.1)	3 (1.4)	0 (0.0)	0.496
Lithiasis	2 (0.7)	2 (0.9)	0 (0.0)	0.627
Stroke	3 (1.1)	2 (0.9)	1 (1.7)	0.504
Tabacco	24 (8.6)	16 (7.2)	8 (13.8)	0.097
Alcohol	64 (22.9)	48 (21.7)	16 (27.6)	0.218
UACR ≥ 30 mg/g	81 (29.0)	58 (26.2)	23 (39.7)	**0.035**
HbF ≥ 15%	46 (18.3)	44 (21.9)	2 (3.9)	**0.001**
HbF < 15%	104 (47.5)	82 (50.0)	22 (40.0)	0.129

Data are expressed as mean ± standard deviation, absolute (n) and relative (in percent) frequencies. Abbreviations: UACR: Urinary albumin to creatinine ratio; FH-VOC: Familial history of vaso-occlusive crisis; HbF: Fetal hemoglobin.

Significant biological differences were also encountered (Table 2). Patients with tubulopathy had higher urinary A1M/Cr (median 44.7 mg/g [27.1–70.8] vs. 14.2 mg/g [7.7–34.6]; *p* < 0.001), UACR (22.1 mg/g [4.6–88.8] vs. 10.7 mg/g [4.1–33.0]; *p* = 0.007). Mean eGFR estimated by the CKD-EPI creatinine–cystatin C 2021 equation was lower in the tubulopathy group (107.4 ± 24.8 vs. 115.9 ± 25.1 mL/min/1.73 m^2^; *p* = 0.031), while measured GFR did not differ significantly (122.5 ± 36.9 vs. 131.8 ± 39.7 mL/min/1.73 m^2^; *p* = 0.180).

**Table 2.** Biological parameters of study population.

Variable	Entire population (*n* = 279)	No tubulopathy (*n* = 221)	Tubulopathy (*n* = 58)	p
Urinary A1M/Cr, mg/g	19.7 (9.6–44.4)	14.2 (7.7–34.6)	44.7 (27.1–70.8)	**<0.001**
Albuminuria, mg/L	18.0 (7.0–57.2)	15.9 (7.7–45.3)	25.9 (4.9–76.7)	0.381
UACR, mg/g	11.2 (4.2–41.5)	10.7 (4.1–33.0)	22.1 (4.6–88.0)	**0.007**
Creatinine, mg/dL	0.6 (0.5–0.7)	0.6 (0.5–0.7)	0.6 (0.5–0.7)	0.671
Cystatin C, mg/L	0.9 (0.7–1.0)	0.8 (0.7–1.0)	0.9 (0.8–1.1)	0.344
mGFR, mL/min/1.73 m^2^	129.7 ± 39.2	131.8 ± 39.7	122.5 ± 36.9	0.180
eGFR, CKD-EPI_crea+cys2021_ (mL/min/1.73 m²)	114.1 ± 25.2	115.9 ± 25.1	107.4 ± 24.8	**0.031**
Urea, mmol/l	4.9 ± 1.7	5.0 ± 1.7	4.7 ± 1.7	0.322
Hb, g/dl	7.5 ± 1.8	7.5 ± 1.8	7.3 ± 1.5	0.480
Hct, %	23.4 ± 5.8	23.6 ± 5.9	22.9 ± 5.3	0.416
RBC, /mm^3^	2.71 ± 0.7	2.73 ± 0.74	2.62 ± 0.67	0.260
Reticulocytes count, %	16.5 ± 2.1	16.4 ± 2.1	16.9 ± 1.7	0.118
WBC, /mm^3^	10.7 (8.8–13.9)	10.6 (8.72–13.55)	11.6 (9.41–14.61)	0.732
Platelets, /mm^3^	161.6 ± 20.9	161.4 ± 22.0	162.4 ± 15.9	0.743
AST, IU/L	33.9 (27.0–45.2)	32.4 (26.4–43.9)	39.2 (30.1–51.8)	**0.002**
Total Bilirubin, mg/dL	1.0 (0.9–1.2)	1.0 (0.9–1.2)	1.0 (0.9–1.2)	0.080
Direct Bilirubin, mg/dL	0.3 (0.2–0.5)	0.3 (0.2–0.5)	0.3 (0.3–0.6)	0.132
LDH, IU/L	210.3 (193.2–265.1)	209.1 (191.3–261.0)	217.0 (196.9–341.6)	**0.016**
Ferritin, ng/mL	267.1 (97.9–663.7)	243.2 (96.3–624.7)	335.8 (138.3–863.8)	0.114
CRP, mg/L	8.5 (3.3–20.3)	8.0 (3.1–19.2)	9.6 (4.8–23.8)	0.222
Urinary pH	6.0 ± 0.43	5.99 ± 0.51	6.08 ± 0.23	0.210

Data are expressed as mean ± standard deviation, median (interquartile range), absolute (n) and relative (in per cent) frequencies. Abbreviations: AST: Aspartate aminotransferase; CRP: C-reactive protein; eGFR: estimated Glomerular filtration rate; Hb: Hemoglobin; Hct: Hematocrit; LDH: Lactate dehydrogenase; RBC: Red blood cells; UACR: Urinary albumin to creatinine ratio; WBC: White blood cells.

No significant differences in hemodynamic or echocardiographic parameters were observed between the two groups (Table 3).

**Table 3.** Hemodynamic and echocardiographic parameters of study population.

Variable	Whole (*n* = 279)	No tubulopathy (*n* = 221)	Tubulopathy (*n* = 58)	p
Age, years	26.3 ± 8.4	26.1 ± 8.2	27.2 ± 9.0	0.356
SBP, mmHg	104.7 ± 13.0	104.9 ± 12.2	103.6 ± 15.6	0.497
DBP, mmHg	58.9 ± 9.9	59.5 ± 9.2	56.9 ± 12.2	0.104
BMI, kg/m^2^	19.2 ± 2.9	19.4 ± 3.0	18.5 ± 2.3	0.050
BSA, m^2^	1.51 ± 0.14	1.51 ± 0.15	1.51 ± 0.12	0.768
PWV, m^2^	5.0 (4.4–5.8)	5.0 (4.4–6.1)	5.0 (4.6–5.5)	0.470
ABI	1.09 ± 0.10	1.09 ± 0.11	1.10 ± 0.08	0.891
IVS, mm	8.7 ± 1.6	8.6 ± 1.5	9.0 ± 1.6	0.272
PW, mm	9.0 ± 1.6	9.0 ± 1.6	9.0 ± 1.5	0.855
LVEF, %	68.0 ± 9.7	67.7 ± 10.0	68.6 ± 8.6	0.615
LVEDD, mm	50.4 ± 5.5	50.4 ± 5.4	50.3 ± 6.1	0.995
RVEDD, mm	28.2 ± 4.9	28.5 ± 4.9	27.1 ± 4.9	0.198
iLVM, g/m^2^	108.1 ± 26.2	107.7 ± 26.5	109.4 ± 25.7	0.767
IVC, mm	12.6 ± 3.1	12.7 ± 3.1	12.3 ± 3.3	0.549
E/A	1.47 ± 0.33	1.5 ± 0.35	1.4 ± 0.23	0.647
E/E’	7.9 ± 1.9	7.9 ± 1.9	7.9 ± 2.1	0.985
TRIV, m/sec	166.6 ± 31.5	166.5 ± 31.2	166.7 ± 32.9	0.973
TAPSE, cm	2.7 ± 0.4	2.7 ± 0.4	2.7 ± 0.3	0.939
Cardiac output, L/’	7.0 (5.5–10.3)	7.0 (5.5–9.7)	7.5 (5.4–25.4)	0.416
SVR, dyn.s/cm^5^	846.6 (686.6–1117.4)	813.7 (668.8–1112.4)	909.0 (762.1–1181.5)	0.682

Data are expressed as mean ± standard deviation, absolute (n) frequencies. Abbreviations: ABI: Ankle brachial index; BMI: Body mass index; DBP: Diastolic blood pressure; SBP: Systolic blood pressure; IVC: Inferior venous cave; IVS: Interventricular septum; LVEDD: Left ventricular end diastolic diameter; LVEF: Left ventricular ejection fraction; iLVM: Indexed left ventricular mass; PW: Posterior wall; PWV: Pulse wave velocity; RVEDD: Right Ventricular end diastolic diameter; TAPSE: Tricuspid annular plane systolic excursion; SVR: Systemic vascular resistance.

**Figure 4.**
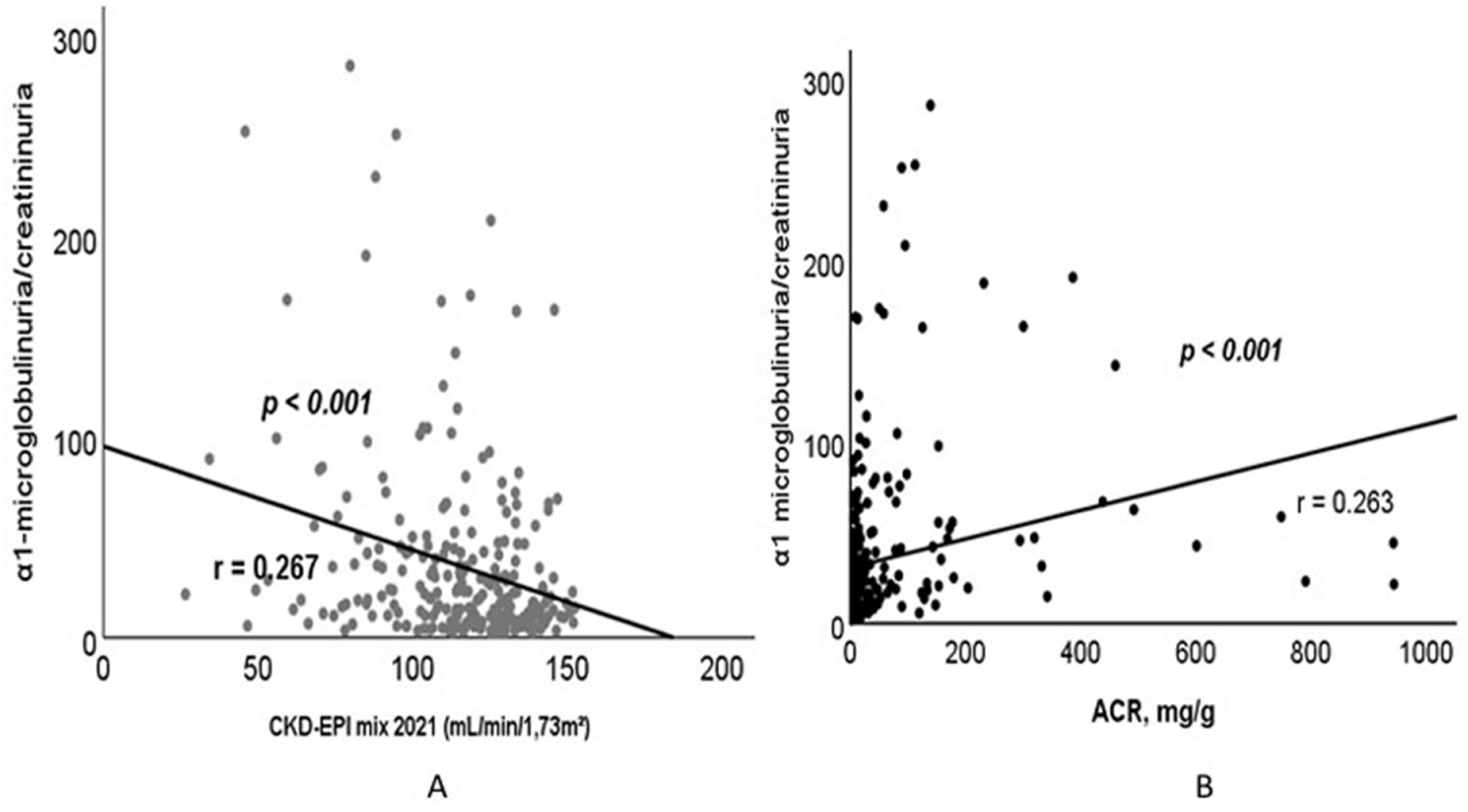
Correlation of eGFR and UACR to AIM/Cr.

Correlation analyses demonstrated significant associations between tubular and glomerular markers. A1M/Cr was inversely correlated with eGFR estimated by the CKD-EPI 2021 creatinine–cystatin C equation (*r* = −0.267, *p* < 0.001) (Figure 4A), indicating that higher tubular proteinuria was observed in patients with lower renal function. In addition, A1M/Cr showed a positive correlation with UACR (*r* = 0.263, *p* < 0.001) (Figure 4B).

In multivariate logistic regression analysis (Table 4), four independent factors were associated with tubulopathy: hydroxyurea use (aOR 0.37, 95% CI 0.16–0.87; *p* = 0.014), higher LDH (per 100 IU/L increase) (aOR 2.53, 95% CI 1.14–5.61; *p* = 0.022), UACR ≥30 mg/g (aOR 1.94, 95% CI 1.18–5.18; *p* = 0.015), and lower eGFR by CKD-EPI creatinine-cystatin C 2021 mL/min/1.73m^2^ (aOR 0.39, 95% CI 0.16–0.95; *p* = 0.016).

**Table 4.** Associated factors to tubulopathy in the study population.

Variables	Univariate analysis		Multivariate analysis	
	p	OR (IC95%)	p	ORa (IC95%)
Hydroxyurea				
No		1		1
Yes	0.018	0.45 (0.23–0.87)	**0.014**	0.37 (0.16–0.87)
Retinopathy				
No		1		1
Yes	0.024	0.25 (0.17–0.83)	0.429	0.69 (0.27–1.74)
UACR ≥ 30 mg/g				
No		1		1
Yes	0.047	1.85 (1.01–5.38)	**0.015**	1.94 (1.18–5.18)
eGFR, CKD-EPIcrea-cys2021 (per 10 mL/min/1.73 m² increase)	0.034	0.39 (0.21–0.99)	**0.016**	0.39 (0.16–0.95)
AST (per 10 IU/L increase)	0.025	1.96 (1.09–3.53)	0.346	1.38 (0.63–3.03)
LDH (per 100 IU/L increase)	0.010	2.67 (1.22–5.82)	**0.022**	2.53 (1.14–5.61)

Abbreviations: eGFR: estimated Glomerular filtration rate; AST: Aspartate aminotransferase; CKD-EPI_crea+cys2021_: Chronic Kidney Disease-Epidemiology combining creatinine -cystatin c; _LDH_: Lactate dehydrogenase; UACR: Urinary albumin to creatinine ratio.

## Discussion

In this multicenter study, approximately one in five adults with sickle cell disease (SCD) in Kinshasa presented with tubular dysfunction despite preserved measured GFR. This prevalence is consistent with recent African reports. In Senegal, Ndour et al. reported frequent glucosuria, α1-microglobulinuria, and abnormal protein/albumin ratios in adults with SCD, confirming that tubular dysfunction is a major but often neglected phenotype in sub-Saharan Africa [6]. Other studies have reported a prevalence of 35-45% based on urinary pH [16], while an Indian study reported even higher prevalences, ranging from 30.4% (hyposthenuria) to 81.3% (abnormal tubular potassium excretion) [20]. However, most of these studies relied on single markers, such as incomplete distal renal tubular acidosis (urine pH >5.3 after acid load) or isolated low-molecular-weight proteinuria. Our stricter composite definition, requiring the concomitant presence of α1-microglobulinuria, glucosuria, and urinary alkalinity (first-morning urine pH ≥5.5), yielded a lower prevalence than studies based on a single tubular marker. Because spot urine pH cannot diagnose impaired distal urinary acidification without metabolic acidosis or standardized acidification testing, this parameter should be interpreted as an exploratory component of the composite phenotype rather than as evidence of distal renal tubular dysfunction. This reinforces the notion that isolated markers may overestimate the burden of clinically significant tubulopathy. Urine pH did not differ significantly between groups. This exploratory component contributed little to discrimination within the composite definition.

Our findings confirm the relevance of α1-microglobulin as a robust marker of proximal tubular injury. Previous studies have shown that elevated α1-microglobulin is consistently associated with albuminuria, hemolysis, and hyperfiltration [7,21,22]. Elsherif et al. [23] reported significantly higher urinary α1-microglobulin in patients with persistent albuminuria, while Saraf et al. [24] demonstrated correlations with KIM-1 and NGAL. Pediatric cohorts also reported that increased α1-microglobulin is linked to early glomerular hyperfiltration and albuminuria [25,26]. The strong association we observed between tubulopathy and albuminuria is biologically plausible. Normally, filtered albumin is reabsorbed by megalin–cubilin receptors in proximal tubules, but this mechanism may be impaired in SCD. Free hemoglobin from hemolysis interferes with albumin uptake, while nitric oxide depletion and endothelin-1 activation exacerbate medullary ischemia and tubular injury [21,22,27,28]. The independent association with ACR ≥30 mg/g observed in our study supports these pathophysiological links. Compared to Western cohorts, where microalbuminuria affects ∼30% of patients [29], our prevalence was higher, though somewhat lower than the ∼25% reported in Saudi Arabia [30], reflecting potential genetic, environmental, and healthcare-related differences.

Higher LDH levels were independently associated with tubulopathy, supporting an association between increasing hemolytic activity and tubular injury in sickle cell disease. Because LDH was analyzed as a continuous variable, this association is less likely to reflect an arbitrary threshold effect and should instead be interpreted as evidence of a graded relationship between hemolysis and tubulopathy. Although an imperfect marker, LDH reflects hemolysis as well as tissue ischemia and inflammation. Hemolysis-mediated release of cell-free hemoglobin and heme promotes oxidative stress, nitric oxide depletion, vasculopathy, and direct proximal tubular toxicity [22,27], a mechanism supported by cortical iron deposition observed in sickle cell disease [31,32]. The lack of association with hemoglobin levels, reticulocyte counts, and aspartate aminotransferase after multivariable adjustment likely reflects steady-state conditions and the limited specificity of these markers. These findings should therefore be interpreted as evidence of statistical association rather than causation, and confirmation in prospective longitudinal studies is warranted [7,33].

Glomerular hyperfiltration was independently associated with lower odds of tubulopathy. This association likely reflects disease stage rather than a biological protective effect, as hyperfiltration predominates in younger patients at earlier stages of sickle cell nephropathy who still have relatively preserved tubular function [34], whereas progressive nephron loss is accompanied by declining GFR and irreversible tubular injury [23,35]. This association should therefore be interpreted cautiously and likely represents temporal bias inherent to the cross-sectional design.

Hydroxyurea therapy has been associated with biological changes that may reduce medullary ischemia and tubular injury, including decreased hemoglobin S polymerization, fewer vaso-occlusive episodes, increased fetal hemoglobin levels, and improved nitric oxide bioavailability [36–38]. In our study, hydroxyurea use was independently associated with lower odds of tubulopathy; however, because treatment duration and adherence were unavailable, this finding should be interpreted cautiously.

This study is the first to assess tubular dysfunction in stable adult SCD patients in the DRC, using iohexol-measured GFR and α1-microglobulin as a robust biomarker and the novel composite definition of tubulopathy. Several limitations should be acknowledged. First, the cross-sectional design precludes causal inference and limits assessment of temporal relationships. Second, tubular markers and albuminuria were measured only once, which may have led to misclassification due to biological variability. Third, recruitment was based on convenience sampling across multiple centers, potentially limiting generalizability. The use of first-morning urine pH ≥5.5 as part of the composite definition was exploratory. Because no metabolic acidosis assessment or standardized acidification test was performed, impaired distal urinary acidification could not be confirmed [16,17]. Finally, detailed data on hydroxyurea exposure (duration, adherence) were not available, constraining interpretation of the observed inverse association between hydroxyurea use and tubulopathy.

## Conclusion

In this multicenter study, nearly one in five stable adults with sickle cell disease in Kinshasa exhibited tubulopathy defined by a stringent multi-marker approach. This phenotype was associated with hemolysis, albuminuria, and lower estimated GFR, while hydroxyurea use was independently associated with lower odds of tubulopathy. These findings underscore the value of tubular screening in sickle cell disease and support prospective longitudinal studies to clarify the temporal relationships underlying these associations and to determine whether disease-modifying therapies influence the progression of tubular dysfunction.

## Data Availability

The data supporting the results of this study are available from the corresponding author, [Yannick Engole], upon reasonable request.
